# (5-Ammonio­pent­yl)triphenyl­phospho­nium dibromide ethanol solvate

**DOI:** 10.1107/S1600536810003193

**Published:** 2010-02-03

**Authors:** Cameron Evans

**Affiliations:** a Department of Chemistry, University of Otago, PO Box 56, Dunedin, New Zealand

## Abstract

The alkyl­ammonium chain of the dication in the title mitochondrially targeted (5-ammonio­pent­yl)triphenyl­­phos­pho­nium dibromide ethanol solvate, C_23_H_28_NP^2+^·2Br^−^·C_2_H_6_O,  is almost planar (r.m.s deviation = 0.0716 Å for all non-H atoms) and in the extended form, maximizing the P⋯N distance [7.716 (2) Å]. The ions and solvent are linked within the crystal by N—H⋯Br, N—H⋯O and O—H⋯Br hydrogen-bonding inter­actions, forming *C*_3_^2^(6) chains along the *b* axis, with secondary C—H⋯Br and C—H⋯O inter­actions cross-linking the chains.

## Related literature

For the development and applications of mitochondrially targeted bio-active compounds, see: Murphy & Smith (2007 ▶); Porteous *et al.* (2010 ▶); Prime *et al.* (2009 ▶). For the synthesis and applications of amino­alkyl triphenyl­phospho­nium salts, see: Issleib & Rieschel (1965 ▶); Keough & Grayson (1964 ▶); McAllister *et al.* (1980 ▶). For related structures, see: Czerwinski (1986 ▶); Dubourg *et al.* (1986 ▶). For a review of hydrogen-bonding networks, see: Bernstein *et al.* (1995 ▶).
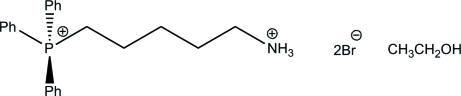


## Experimental

### 

#### Crystal data

C_23_H_28_NP^2+^·2Br^−^·C_2_H_6_O
*M* *_r_* = 555.32
Orthorhombic, 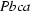

*a* = 16.600 (3) Å
*b* = 11.947 (2) Å
*c* = 26.257 (5) Å
*V* = 5207.3 (18) Å^3^
*Z* = 8
Mo *K*α radiation
μ = 3.19 mm^−1^
*T* = 89 K
0.27 × 0.25 × 0.25 mm

#### Data collection

Bruker APEXII CCD area-detector diffractometer
Absorption correction: multi-scan (*SADABS*; Bruker, 2006 ▶) *T*_min_ = 0.379, *T*_max_ = 0.450
98142 measured reflections
5323 independent reflections
4470 reflections with *I* > 2σ(*I*)
*R*_int_ = 0.063

#### Refinement

*R*[*F* ^2^ > 2σ(*F* ^2^)] = 0.024
*wR*(*F* ^2^) = 0.056
*S* = 1.04
5323 reflections
274 parameters
H-atom parameters constrained
Δρ_max_ = 0.37 e Å^−3^
Δρ_min_ = −0.33 e Å^−3^

### 

Data collection: *APEX2* (Bruker, 2006 ▶); cell refinement: *APEX2* and *SAINT* (Bruker, 2006 ▶); data reduction: *SAINT*; program(s) used to solve structure: *SIR97* (Altomare *et al.*, 1999 ▶); program(s) used to refine structure: *SHELXL97* (Sheldrick, 2008 ▶); molecular graphics: *ORTEP-3 for Windows* (Farrugia, 1997 ▶) and *Mercury* (Macrae *et al.*, 2006 ▶).; software used to prepare material for publication: *WinGX* (Farrugia, 1999 ▶), *enCIFer* (Allen *et al.*, 2004 ▶) and *publCIF* (Westrip, 2010 ▶).

## Supplementary Material

Crystal structure: contains datablocks I, global. DOI: 10.1107/S1600536810003193/nc2175sup1.cif

Structure factors: contains datablocks I. DOI: 10.1107/S1600536810003193/nc2175Isup2.hkl

Additional supplementary materials:  crystallographic information; 3D view; checkCIF report

## Figures and Tables

**Table 1:** Hydrogen-bond geometry (Å, °)

*D*—H⋯*A*	*D*—H	H⋯*A*	*D*⋯*A*	*D*—H⋯*A*
N1—H1*C*⋯O1^i^	0.89	1.9	2.790 (3)	177
N1—H1*D*⋯Br1^ii^	0.89	2.34	3.2226 (18)	170
O1—H1⋯Br2	0.82	2.43	3.2397 (16)	170
N1—H1*E*⋯Br2	0.89	2.4	3.2814 (18)	168
C13—H13⋯O1^iii^	0.93	2.66	3.584 (3)	172
C34—H34⋯Br2^iv^	0.93	3	3.729 (2)	137
C1—H1*A*⋯Br1^v^	0.97	2.92	3.836 (2)	159
C99—H99*B*⋯Br1^v^	0.96	2.92	3.849 (3)	163
C1—H1*B*⋯Br1	0.97	2.92	3.886 (2)	173

Symmetry codes: (i) 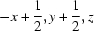; (ii) 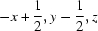; (iii) 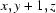; (iv) 
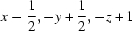
; (v) 
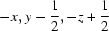
.

**Fig. 1.**
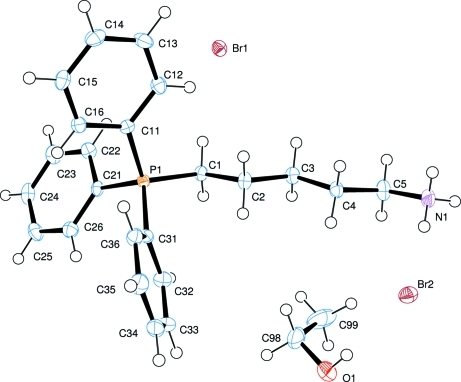
The asymmetric unit showing the atom labelling scheme. Ellipsoids are drawn at the 50% probability level with H atoms represented by spheres of arbitrary size.

**Fig. 2.**
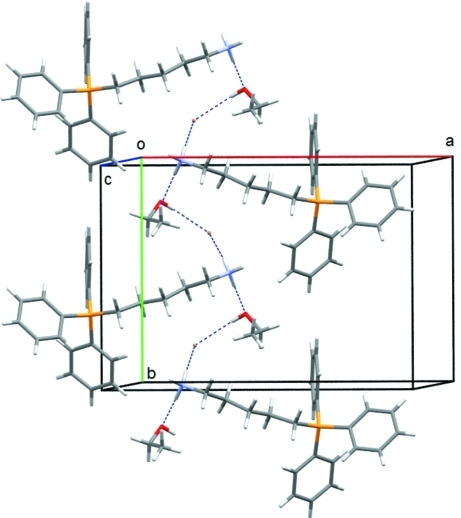
The dominant hydrogen-bond contacts drawn as dashed lines forming C23(6) chains along the b axis.

## supplementary crystallographic information

### Comment

In the course of our research in mitochondrially targeted bio-active agents (Murphy & Smith, 2007), 5-ammoniopentyl triphenylphosphonium dibromide was prepared as a precursor for both a targeted thionitrite (Prime *et al.*, 2009) and an iodoacetamide (Porteous *et al.*, 2010). Aminoalkyl-triphenylphosphonium salts are well known (McAllister *et al.*, 1980; Keough & Grayson, 1964; Issleib & Rieschel, 1965) but to date only dimethylamino-3-propyl triphenylphosphonium chloride (Dubourg *et al.*, 1986) and 2-aminoethyl triphenylphosphonium bromide hydrogen bromide (Czerwinski, 1986) have been structurally characterized. The latter is clearly a dication with two bromide anions whereas the former contains a singly charged aminoalkyl triphenylphosphonium cation.

The asymmetric unit (Fig. 1) of the title compound contains one dication, two bromide ions and an ethanol of cystallization. The C(1)—P(1) and C(5)—N(1) distances [1.8020 (19)Å and 1.496 (3)Å respectively] are comparable to those published for the singly charged dimethylamino-propyl [1.802 (3)Å and 1.496 (9) Å; Dubourg *et al.*, 1986] and dicationic 2-aminoethyl [1.796 (5)Å and 1.512 (6) Å; Czerwinski, 1986] structures, indicating that the charge and increased alkyl chain length have no appreciable effect on these parameters. The P(1)···N(1) chain is almost planar [r.m.s for all non-H atoms of 0.0716] and is in the extended form as indicated by the P—C, C—C, and C—N torsion angles [P—C(1)—C(2)—C(3) 178.55 (15)°, C(1)—C(2)—C(3)—C(4) -172.83 (17)°,*C*(2)—C(3)—C(4)—C(5) 173.97 (18)°, C(3)—C(4)—C(5)—N(1) 172.94 (17)°].

The hydrogen bonding network is dominated by a C^2^_3_(6) chain motif (Bernstein *et al.*, 1995) linking the dication, Br(2) and the solvent molecule (Fig. 2). The chains are comprised of N(1)—H(1C)···O(1)—H(1)···Br(2)···H(1E) interactions [N(1)···O(1) 2.790 (3) Å, O(1)···Br(2) 3.2397 (16) Å, N(1)···Br(2) 3.2814 (18) Å] which form linkages along the *b* axis. These chains are in turn cross-linked at longer distances by C(13)—H(13)···O(1) [C(13)···O(1) 3.584 (3) Å] and C(34)—H(34)···Br(2) [C(34)···Br(2) 3.729 (2) Å] interactions. In addition, Br(1) adopts an approximately square planar arrangement (r.m.s 0.2377) with contacts to C(1) in a *trans* configuration linking adjacent dications [C(1)···Br(1) 3.836 (2) Å, C(1)···Br(1) 3.886 (2) Å], and contacts to N(1)—H(1D) [N(1)···Br(1) 3.2226 (18) Å] and C(99)—H99(B) [C(99)···Br(1) 3.849 (3) Å] linking the dication and solvent.

### Experimental

The title compound was prepared using a modified procedure to that published (McAllister *et al.*, 1980). Crystalline 5-bromopentyl-amine hydrogen bromide (3 mmol) and excess triphenylphosphine (9 mmol) were heated under an inert atmosphere to 100°C and stirred for 72 h. After cooling to room temperature, the mixture was dissolved in ethanol (5 mL) and added to diethyl ether (20 mL) to precipitate the compound. Crystals of suitable quality were grown by diffusion of diethylether into an ethanolic solution of the compound.

### Refinement

All H-atoms were positioned geometrically (OH allowed to rotate but not to tip) and refined using a riding model with d(C—H) = 0.93 Å, *U*_iso_=1.2*U*_eq_ (C) for aromatic 0.97 Å, *U*_iso_ = 1.2*U*_eq_ (C) for CH_2_ 0.96 Å, *U*_iso_ = 1.5*U*_eq_ (C) for CH_3_ 0.89 Å, *U*_iso_ = 1.5*U*_eq_ (N) for the NH_3_ atoms and 0.82Å, *U*_iso_ = *U*1.5_eq_ (O) for the OH atoms.

### Crystal data

**Table:**

C_23_H_28_NP^2+^·2Br^−^·C_2_H_6_O	*F*(000) = 2272
*M**_r_* = 555.32	*D*_x_ = 1.417 Mg m^−^^3^
Orthorhombic, *P**b**c**a*	Mo *K*α radiation, λ = 0.71073 Å
Hall symbol: -P 2ac 2ab	Cell parameters from 8129 reflections
*a* = 16.600 (3) Å	θ = 2.6–26.2°
*b* = 11.947 (2) Å	µ = 3.19 mm^−^^1^
*c* = 26.257 (5) Å	*T* = 89 K
*V* = 5207.3 (18) Å^3^	Prism, colourless
*Z* = 8	0.27 × 0.25 × 0.25 mm

### Data collection

**Table:**

Bruker APEXII CCD area-detector diffractometer	5323 independent reflections
Radiation source: fine-focus sealed tube	4470 reflections with *I* > 2σ(*I*)
graphite	*R*_int_ = 0.063
φ and ω scans	θ_max_ = 26.4°, θ_min_ = 1.6°
Absorption correction: multi-scan (*SADABS*; Bruker, 2006)	*h* = −20→20
*T*_min_ = 0.379, *T*_max_ = 0.450	*k* = −14→14
98142 measured reflections	*l* = −32→32

### Refinement

**Table:**

Refinement on *F*^2^	Primary atom site location: structure-invariant direct methods
Least-squares matrix: full	Secondary atom site location: difference Fourier map
*R*[*F*^2^ > 2σ(*F*^2^)] = 0.024	Hydrogen site location: inferred from neighbouring sites
*wR*(*F*^2^) = 0.056	H-atom parameters constrained
*S* = 1.04	*w* = 1/[σ^2^(*F*_o_^2^) + (0.0205*P*)^2^ + 4.4181*P*] where *P* = (*F*_o_^2^ + 2*F*_c_^2^)/3
5323 reflections	(Δ/σ)_max_ = 0.001
274 parameters	Δρ_max_ = 0.37 e Å^−^^3^
0 restraints	Δρ_min_ = −0.33 e Å^−^^3^

### Special details

**Table:**

Geometry. All e.s.d.'s (except the e.s.d. in the dihedral angle between two l.s. planes) are estimated using the full covariance matrix. The cell e.s.d.'s are taken into account individually in the estimation of e.s.d.'s in distances, angles and torsion angles; correlations between e.s.d.'s in cell parameters are only used when they are defined by crystal symmetry. An approximate (isotropic) treatment of cell e.s.d.'s is used for estimating e.s.d.'s involving l.s. planes.
Refinement. Refinement of *F*^2^ against ALL reflections. The weighted *R*-factor *wR* and goodness of fit *S* are based on *F*^2^, conventional *R*-factors *R* are based on *F*, with *F* set to zero for negative *F*^2^. The threshold expression of *F*^2^ > σ(*F*^2^) is used only for calculating *R*-factors(gt) *etc*. and is not relevant to the choice of reflections for refinement. *R*-factors based on *F*^2^ are statistically about twice as large as those based on *F*, and *R*- factors based on ALL data will be even larger.

### Fractional atomic coordinates and isotropic or equivalent isotropic displacement parameters (Å^2^)

**Table:**

	*x*	*y*	*z*	*U*_iso_*/*U*_eq_	
C1	−0.03376 (11)	0.67164 (17)	0.33559 (7)	0.0157 (4)	
H1A	−0.048	0.6174	0.3096	0.019*	
H1B	−0.0242	0.7428	0.3189	0.019*	
C2	0.04374 (12)	0.63369 (19)	0.36243 (8)	0.0193 (4)	
H2A	0.0584	0.6889	0.3879	0.023*	
H2B	0.0335	0.5636	0.3799	0.023*	
C3	0.11389 (12)	0.61792 (18)	0.32598 (7)	0.0187 (4)	
H3A	0.1292	0.6898	0.3118	0.022*	
H3B	0.0976	0.5696	0.2982	0.022*	
C4	0.18562 (12)	0.56646 (17)	0.35333 (8)	0.0184 (4)	
H4A	0.2049	0.6186	0.3789	0.022*	
H4B	0.1683	0.499	0.3707	0.022*	
C5	0.25410 (13)	0.5377 (2)	0.31770 (8)	0.0248 (5)	
H5A	0.2376	0.4781	0.2949	0.03*	
H5B	0.2678	0.6026	0.2973	0.03*	
C11	−0.10746 (12)	0.81365 (16)	0.41553 (7)	0.0145 (4)	
C12	−0.04349 (12)	0.88679 (18)	0.40813 (8)	0.0200 (5)	
H12	−0.0024	0.8686	0.3855	0.024*	
C13	−0.04125 (13)	0.98715 (19)	0.43472 (8)	0.0249 (5)	
H13	0.0012	1.0367	0.4297	0.03*	
C14	−0.10222 (13)	1.01350 (19)	0.46868 (8)	0.0241 (5)	
H14	−0.1001	1.0805	0.4866	0.029*	
C15	−0.16599 (13)	0.94130 (18)	0.47611 (8)	0.0224 (5)	
H15	−0.2068	0.9599	0.4989	0.027*	
C16	−0.16931 (12)	0.84124 (17)	0.44969 (8)	0.0190 (4)	
H16	−0.2123	0.7925	0.4546	0.023*	
C21	−0.21088 (11)	0.69250 (17)	0.34621 (7)	0.0155 (4)	
C22	−0.22483 (13)	0.78484 (18)	0.31458 (8)	0.0210 (5)	
H22	−0.1846	0.8378	0.3095	0.025*	
C23	−0.29898 (13)	0.7969 (2)	0.29092 (8)	0.0244 (5)	
H23	−0.3087	0.8586	0.2702	0.029*	
C24	−0.35848 (13)	0.71784 (19)	0.29802 (8)	0.0234 (5)	
H24	−0.4082	0.7269	0.2822	0.028*	
C25	−0.34490 (13)	0.62523 (19)	0.32838 (8)	0.0241 (5)	
H25	−0.3848	0.5713	0.3323	0.029*	
C26	−0.27107 (12)	0.61296 (18)	0.35303 (8)	0.0216 (5)	
H26	−0.2621	0.5516	0.3741	0.026*	
C31	−0.11586 (11)	0.56603 (16)	0.42049 (8)	0.0162 (4)	
C32	−0.11609 (13)	0.45969 (18)	0.39805 (8)	0.0225 (5)	
H32	−0.1173	0.4528	0.3628	0.027*	
C33	−0.11454 (13)	0.36506 (19)	0.42816 (9)	0.0266 (5)	
H33	−0.1156	0.2945	0.4132	0.032*	
C34	−0.11135 (13)	0.3750 (2)	0.48077 (9)	0.0286 (5)	
H34	−0.1095	0.3112	0.501	0.034*	
C35	−0.11098 (13)	0.47944 (19)	0.50297 (8)	0.0270 (5)	
H35	−0.1094	0.4856	0.5383	0.032*	
C36	−0.11300 (12)	0.57552 (18)	0.47341 (8)	0.0206 (5)	
H36	−0.1125	0.6457	0.4887	0.025*	
N1	0.32613 (10)	0.50107 (16)	0.34763 (7)	0.0234 (4)	
H1C	0.3441	0.558	0.3663	0.035*	
H1D	0.3647	0.4787	0.3264	0.035*	
H1E	0.3124	0.4447	0.368	0.035*	
P1	−0.11631 (3)	0.68613 (4)	0.379672 (19)	0.01355 (11)	
Br1	0.026856 (12)	0.951555 (17)	0.270710 (8)	0.01980 (6)	
Br2	0.273685 (13)	0.321417 (17)	0.436159 (8)	0.02177 (6)	
O1	0.11446 (9)	0.18279 (13)	0.40281 (7)	0.0322 (4)	
H1	0.1543	0.2138	0.4147	0.048*	
C98	0.07010 (15)	0.2606 (2)	0.37272 (13)	0.0492 (8)	
H98A	0.0694	0.3324	0.3899	0.059*	
H98B	0.0149	0.2349	0.3699	0.059*	
C99	0.1044 (2)	0.2760 (2)	0.31995 (12)	0.0539 (8)	
H99A	0.1601	0.2971	0.3224	0.081*	
H99B	0.075	0.3335	0.3025	0.081*	
H99C	0.1	0.207	0.3014	0.081*	

### Atomic displacement parameters (Å^2^)

**Table:**

	*U*^11^	*U*^22^	*U*^33^	*U*^12^	*U*^13^	*U*^23^
C1	0.0163 (10)	0.0185 (11)	0.0124 (9)	0.0011 (8)	0.0016 (8)	−0.0001 (8)
C2	0.0157 (10)	0.0247 (11)	0.0174 (10)	0.0028 (9)	−0.0001 (8)	−0.0004 (9)
C3	0.0181 (10)	0.0232 (11)	0.0147 (10)	0.0009 (9)	0.0008 (8)	−0.0014 (9)
C4	0.0156 (10)	0.0211 (11)	0.0186 (10)	0.0001 (8)	0.0014 (8)	−0.0027 (9)
C5	0.0172 (10)	0.0341 (13)	0.0232 (11)	0.0031 (10)	0.0028 (9)	0.0005 (10)
C11	0.0168 (10)	0.0140 (10)	0.0128 (9)	0.0009 (8)	−0.0026 (8)	0.0000 (8)
C12	0.0199 (11)	0.0232 (12)	0.0171 (10)	−0.0005 (9)	0.0033 (8)	−0.0002 (9)
C13	0.0259 (12)	0.0209 (12)	0.0277 (12)	−0.0086 (9)	0.0014 (10)	−0.0008 (9)
C14	0.0304 (12)	0.0192 (11)	0.0226 (12)	0.0015 (10)	−0.0053 (10)	−0.0065 (9)
C15	0.0211 (11)	0.0259 (12)	0.0203 (11)	0.0048 (9)	0.0023 (9)	−0.0041 (9)
C16	0.0158 (10)	0.0193 (11)	0.0219 (11)	0.0000 (9)	−0.0017 (9)	0.0000 (9)
C21	0.0135 (9)	0.0192 (11)	0.0139 (10)	0.0015 (8)	−0.0004 (8)	−0.0032 (8)
C22	0.0199 (11)	0.0235 (11)	0.0195 (11)	−0.0004 (9)	−0.0004 (9)	0.0020 (9)
C23	0.0224 (11)	0.0324 (13)	0.0184 (11)	0.0040 (10)	−0.0020 (9)	0.0050 (10)
C24	0.0164 (10)	0.0393 (14)	0.0145 (10)	0.0052 (10)	−0.0022 (8)	−0.0058 (10)
C25	0.0168 (11)	0.0310 (13)	0.0244 (12)	−0.0057 (9)	−0.0005 (9)	−0.0050 (10)
C26	0.0213 (11)	0.0218 (11)	0.0216 (11)	0.0001 (9)	−0.0001 (9)	−0.0004 (9)
C31	0.0133 (9)	0.0166 (10)	0.0187 (10)	−0.0005 (8)	0.0005 (8)	0.0035 (8)
C32	0.0240 (11)	0.0210 (11)	0.0227 (11)	−0.0016 (9)	−0.0007 (9)	0.0000 (9)
C33	0.0250 (12)	0.0166 (11)	0.0383 (14)	−0.0019 (9)	0.0026 (10)	0.0014 (10)
C34	0.0232 (12)	0.0263 (13)	0.0362 (14)	0.0029 (10)	0.0065 (10)	0.0155 (11)
C35	0.0285 (12)	0.0316 (14)	0.0209 (11)	0.0051 (10)	0.0045 (10)	0.0088 (10)
C36	0.0202 (10)	0.0214 (11)	0.0203 (11)	0.0027 (9)	0.0017 (9)	0.0006 (9)
N1	0.0164 (9)	0.0255 (10)	0.0283 (10)	0.0027 (8)	0.0072 (8)	0.0017 (8)
P1	0.0129 (2)	0.0145 (3)	0.0132 (2)	0.0000 (2)	−0.00030 (19)	0.0003 (2)
Br1	0.01722 (10)	0.02281 (11)	0.01939 (11)	−0.00049 (8)	−0.00321 (8)	0.00172 (9)
Br2	0.02484 (12)	0.01773 (11)	0.02274 (11)	0.00034 (9)	−0.00399 (9)	−0.00094 (9)
O1	0.0239 (9)	0.0302 (9)	0.0424 (10)	−0.0056 (7)	−0.0007 (7)	−0.0017 (8)
C98	0.0217 (13)	0.0303 (15)	0.095 (3)	0.0039 (11)	−0.0116 (15)	−0.0074 (15)
C99	0.067 (2)	0.0290 (15)	0.066 (2)	0.0049 (14)	−0.0318 (17)	0.0120 (14)

### Geometric parameters (Å, °)

**Table:**

C1—C2	1.535 (3)	C22—H22	0.93
C1—P1	1.8020 (19)	C23—C24	1.380 (3)
C1—H1A	0.97	C23—H23	0.93
C1—H1B	0.97	C24—C25	1.382 (3)
C2—C3	1.519 (3)	C24—H24	0.93
C2—H2A	0.97	C25—C26	1.394 (3)
C2—H2B	0.97	C25—H25	0.93
C3—C4	1.520 (3)	C26—H26	0.93
C3—H3A	0.97	C31—C36	1.395 (3)
C3—H3B	0.97	C31—C32	1.400 (3)
C4—C5	1.512 (3)	C31—P1	1.791 (2)
C4—H4A	0.97	C32—C33	1.380 (3)
C4—H4B	0.97	C32—H32	0.93
C5—N1	1.496 (3)	C33—C34	1.387 (3)
C5—H5A	0.97	C33—H33	0.93
C5—H5B	0.97	C34—C35	1.377 (3)
C11—C12	1.389 (3)	C34—H34	0.93
C11—C16	1.403 (3)	C35—C36	1.386 (3)
C11—P1	1.797 (2)	C35—H35	0.93
C12—C13	1.388 (3)	C36—H36	0.93
C12—H12	0.93	N1—H1C	0.89
C13—C14	1.385 (3)	N1—H1D	0.89
C13—H13	0.93	N1—H1E	0.89
C14—C15	1.379 (3)	O1—C98	1.425 (3)
C14—H14	0.93	O1—H1	0.82
C15—C16	1.383 (3)	C98—C99	1.509 (4)
C15—H15	0.93	C98—H98A	0.97
C16—H16	0.93	C98—H98B	0.97
C21—C26	1.391 (3)	C99—H99A	0.96
C21—C22	1.400 (3)	C99—H99B	0.96
C21—P1	1.801 (2)	C99—H99C	0.96
C22—C23	1.386 (3)		
			
C2—C1—P1	111.77 (13)	C24—C23—H23	119.9
C2—C1—H1A	109.3	C22—C23—H23	119.9
P1—C1—H1A	109.3	C23—C24—C25	120.6 (2)
C2—C1—H1B	109.3	C23—C24—H24	119.7
P1—C1—H1B	109.3	C25—C24—H24	119.7
H1A—C1—H1B	107.9	C24—C25—C26	119.7 (2)
C3—C2—C1	112.95 (16)	C24—C25—H25	120.1
C3—C2—H2A	109	C26—C25—H25	120.1
C1—C2—H2A	109	C21—C26—C25	120.0 (2)
C3—C2—H2B	109	C21—C26—H26	120
C1—C2—H2B	109	C25—C26—H26	120
H2A—C2—H2B	107.8	C36—C31—C32	119.53 (19)
C2—C3—C4	110.66 (16)	C36—C31—P1	122.08 (16)
C2—C3—H3A	109.5	C32—C31—P1	118.36 (15)
C4—C3—H3A	109.5	C33—C32—C31	120.1 (2)
C2—C3—H3B	109.5	C33—C32—H32	119.9
C4—C3—H3B	109.5	C31—C32—H32	119.9
H3A—C3—H3B	108.1	C32—C33—C34	120.1 (2)
C5—C4—C3	112.86 (17)	C32—C33—H33	120
C5—C4—H4A	109	C34—C33—H33	120
C3—C4—H4A	109	C35—C34—C33	120.0 (2)
C5—C4—H4B	109	C35—C34—H34	120
C3—C4—H4B	109	C33—C34—H34	120
H4A—C4—H4B	107.8	C34—C35—C36	120.9 (2)
N1—C5—C4	110.02 (17)	C34—C35—H35	119.6
N1—C5—H5A	109.7	C36—C35—H35	119.6
C4—C5—H5A	109.7	C35—C36—C31	119.4 (2)
N1—C5—H5B	109.7	C35—C36—H36	120.3
C4—C5—H5B	109.7	C31—C36—H36	120.3
H5A—C5—H5B	108.2	C5—N1—H1C	109.5
C12—C11—C16	120.08 (18)	C5—N1—H1D	109.5
C12—C11—P1	121.51 (15)	H1C—N1—H1D	109.5
C16—C11—P1	118.33 (15)	C5—N1—H1E	109.5
C13—C12—C11	119.58 (19)	H1C—N1—H1E	109.5
C13—C12—H12	120.2	H1D—N1—H1E	109.5
C11—C12—H12	120.2	C31—P1—C11	111.43 (9)
C14—C13—C12	120.0 (2)	C31—P1—C21	109.24 (9)
C14—C13—H13	120	C11—P1—C21	106.92 (9)
C12—C13—H13	120	C31—P1—C1	107.71 (9)
C15—C14—C13	120.6 (2)	C11—P1—C1	110.84 (9)
C15—C14—H14	119.7	C21—P1—C1	110.71 (9)
C13—C14—H14	119.7	C98—O1—H1	109.5
C14—C15—C16	120.0 (2)	O1—C98—C99	113.2 (2)
C14—C15—H15	120	O1—C98—H98A	108.9
C16—C15—H15	120	C99—C98—H98A	108.9
C15—C16—C11	119.65 (19)	O1—C98—H98B	108.9
C15—C16—H16	120.2	C99—C98—H98B	108.9
C11—C16—H16	120.2	H98A—C98—H98B	107.8
C26—C21—C22	119.74 (19)	C98—C99—H99A	109.5
C26—C21—P1	122.32 (16)	C98—C99—H99B	109.5
C22—C21—P1	117.84 (15)	H99A—C99—H99B	109.5
C23—C22—C21	119.7 (2)	C98—C99—H99C	109.5
C23—C22—H22	120.2	H99A—C99—H99C	109.5
C21—C22—H22	120.2	H99B—C99—H99C	109.5
C24—C23—C22	120.2 (2)		
			
P1—C1—C2—C3	178.55 (15)	C34—C35—C36—C31	0.3 (3)
C1—C2—C3—C4	−172.83 (17)	C32—C31—C36—C35	−0.4 (3)
C2—C3—C4—C5	173.97 (18)	P1—C31—C36—C35	−178.57 (16)
C3—C4—C5—N1	172.94 (17)	C36—C31—P1—C11	3.0 (2)
C16—C11—C12—C13	0.0 (3)	C32—C31—P1—C11	−175.18 (16)
P1—C11—C12—C13	176.62 (16)	C36—C31—P1—C21	−114.94 (17)
C11—C12—C13—C14	0.5 (3)	C32—C31—P1—C21	66.91 (18)
C12—C13—C14—C15	−0.6 (3)	C36—C31—P1—C1	124.75 (17)
C13—C14—C15—C16	0.3 (3)	C32—C31—P1—C1	−53.40 (18)
C14—C15—C16—C11	0.2 (3)	C12—C11—P1—C31	119.73 (17)
C12—C11—C16—C15	−0.3 (3)	C16—C11—P1—C31	−63.56 (18)
P1—C11—C16—C15	−177.07 (15)	C12—C11—P1—C21	−120.97 (17)
C26—C21—C22—C23	0.9 (3)	C16—C11—P1—C21	55.74 (18)
P1—C21—C22—C23	−175.53 (16)	C12—C11—P1—C1	−0.22 (19)
C21—C22—C23—C24	−0.7 (3)	C16—C11—P1—C1	176.49 (15)
C22—C23—C24—C25	−0.5 (3)	C26—C21—P1—C31	−0.2 (2)
C23—C24—C25—C26	1.5 (3)	C22—C21—P1—C31	176.17 (15)
C22—C21—C26—C25	0.1 (3)	C26—C21—P1—C11	−120.89 (17)
P1—C21—C26—C25	176.38 (16)	C22—C21—P1—C11	55.46 (18)
C24—C25—C26—C21	−1.3 (3)	C26—C21—P1—C1	118.28 (17)
C36—C31—C32—C33	0.8 (3)	C22—C21—P1—C1	−65.38 (18)
P1—C31—C32—C33	179.03 (16)	C2—C1—P1—C31	−44.82 (17)
C31—C32—C33—C34	−1.1 (3)	C2—C1—P1—C11	77.32 (16)
C32—C33—C34—C35	1.0 (3)	C2—C1—P1—C21	−164.20 (14)
C33—C34—C35—C36	−0.6 (3)		

### Hydrogen-bond geometry (Å, °)

**Table:**

*D*—H···*A*	*D*—H	H···*A*	*D*···*A*	*D*—H···*A*
N1—H1C···O1^i^	0.89	1.9	2.790 (3)	177
N1—H1D···Br1^ii^	0.89	2.34	3.2226 (18)	170
O1—H1···Br2	0.82	2.43	3.2397 (16)	170
N1—H1E···Br2	0.89	2.4	3.2814 (18)	168
C13—H13···O1^iii^	0.93	2.66	3.584 (3)	172
C34—H34···Br2^iv^	0.93	3	3.729 (2)	137
C1—H1A···Br1^v^	0.97	2.92	3.836 (2)	159
C99—H99B···Br1^v^	0.96	2.92	3.849 (3)	163
C1—H1B···Br1	0.97	2.92	3.886 (2)	173

Symmetry codes: (i) −*x*+1/2, *y*+1/2, *z*; (ii) −*x*+1/2, *y*−1/2, *z*; (iii) *x*, *y*+1, *z*; (iv) *x*−1/2, −*y*+1/2, −*z*+1; (v) −*x*, *y*−1/2, −*z*+1/2.
